# Abdominoplasty after massive weight loss. Safety preservation fascia technique and clinical outcomes in a large single series-comparative study

**DOI:** 10.3389/fsurg.2024.1337948

**Published:** 2024-01-25

**Authors:** Claudio Cannistrà, Eleonora Lori, Konstantinos Arapis, Gaetano Gallo, Marzia Varanese, Daniele Pironi, Alessandro De Luca, Federico Frusone, Maria Ida Amabile, Salvatore Sorrenti, Federica Gagliardi, Domenico Tripodi

**Affiliations:** ^1^ Plastic and Reconstructive Surgery Unit, Centre Hospitalier Universitaire Bichat Claude-Bernard, Paris, France; ^2^ Department of Surgical, Sapienza University of Rome, Rome, Italy

**Keywords:** bariatric surgery, body contouring surgery, seroma, Scarpa fascia preservation, abdominoplasty

## Abstract

**Introduction:**

Weight loss after bariatric surgery causes very important modifications to the patient's silhouette. Abdominal fat and skin excess reduction are associated with several complications. The most frequent are seroma and hematoma whereas major complications, such as pulmonary embolism, are less frequent. This study aimed to describe our technical procedure for abdominoplasty in patients with massive weight loss after bariatric surgery.

**Methods:**

In total, 196 patients were included. All patients who underwent abdominoplasty classic (group A) and abdominoplasty with the preservation and lift of Scarpa fascia (group B) and with umbilical transposition between May 2018 and May 2021 were included. Patients with concomitant correction of ventral hernia were excluded. Demographic and operative data were analyzed according to comorbidities and postoperative complications.

**Results:**

There were 160 (81.6%) women. The mean age was 43.6 years; the mean weight was 86.7 kg; and the mean BMI was 28.6 kg/m^2^. Five patients (2.5%) presented postoperative seroma. Four patients (2%) presented partial dehiscence/skin necrosis one of them requiring a revision. Finally, 26 patients presented a postoperative complication, with an overall incidence of 12.6%. The average postoperative hospital stay was 3.6. The rates of seroma were significantly higher in men, patients with a BMI > 30 kg/m^2^, and aged >50 years.

**Conclusion:**

Preserving Scarpa Fascia during surgical post-bariatric patient procedures reduces the seroma formation and the scar complication and reduces the tension of the inguinal-pubic region with correction of our deformation after weight loss. Improves reducing the drain and reducing seroma incidence suction and hospital stay.

## 1 Introduction

Weight loss after bariatric surgery causes very important modifications to patients’ silhouettes. Abdominoplasty still presents high complication rates (1–5) after surgical removal of excess skin from the abdominal wall after massive weight loss. The most common complications are seroma (30%), hematoma (10%), wound dehiscence or infection (50%), whereas major complications such as pulmonary embolism, deep vein thrombosis (6), and postoperative anemia are less frequent (1%). Complications are more frequent in people with obesity than in non-obese (7, 8). Hematoma is an early severe complication, which can sometimes be life-threatening, especially in post-bariatric patients**.** Seroma is a complication that arise later. Although not life-threatening, seromas can cause patients great discomfort. Innovations in abdominoplasty include Scarpa fascia preservation for lowering the seroma rate and reducing the volume and duration of the drain. The study aims to compare a technical procedure to decrease the rate of postoperative complications and the technical aspect of preservation and lifting of the Scarpa fascia during abdominoplasty.

## 2 Materials and methods

### 2.1 Methods

The present study is a retrospective single-center study and is reported according to the Strengthening the Reporting of Observational Studies in Epidemiology (STROBE) statement for cohort studies (9), conducted in Paris, France, at Hospital Bichat-Claude Bernard. After Institutional review board and ethical committee approval, the medical records of all the patients who underwent abdominoplasty with umbilical transposition, between May 2018 and May 2021, in the Unit of Plastic Surgery of the Department of General and Visceral Surgery were consulted. Data were summarized descriptively. All surgical procedures were performed by experienced and high-volume surgeons, with large experience in plastic post bariatric surgery. All the patients had a history of bariatric surgery for morbid obesity. At the time of the bariatric procedure, all the patients met the National Institute of Health criteria (10) for bariatric surgery and were screened preoperatively by a multidisciplinary team including a clinical nutritionist, psychiatrist, clinical psychologist, and surgeon. All the patients were followed up four times during the first year after the bariatric operation and twice a year thereafter. Consultation with a plastic surgeon was proposed up to two years after the operation if weight stability, defined as Body Mass Index (BMI) < 32 kg/m^2^ for at least 6 months (11), was achieved. Exclusion criteria were as follows: elevated operative health risks, and patients who anticipate future pregnancy. The smokers are instructed to reduce smoking 6 days before and after surgery. Patients were excluded from this analysis if they underwent a ventral hernia operation simultaneously or had a history of previous abdominal contouring procedures. A nutritional assessment, including measurements of albumin and prealbumin levels, iron, and vitamins A, D, E, K, B1, B6, and B12, was performed. Corrective measures were taken in case of deficiency before reconstructive surgery. Each patient received preoperative care including enoxaparin 40 mg/day subcutaneously starting at least 2 h before surgery for 1 week and broad-spectrum intravenous antibiotics, wearing elastic compression stockings during recovery. The patients were allocated to one of two different procedures: classic abdominoplasty (group A) and Scarpa Fascia preservation abdominoplasty (group B). Data regarding patient sex, age, BMI, percentage of excess body weight loss (EWL %), and operative variables (operative time, transfusion, postoperative complications, and reoperations). EWL % and ideal body weight (IBW) were calculated using the following equations (12) EWL %: [(preoperative weight (Kg)—current weight (Kg))/(preoperative weight (Kg)—ideal weight (Kg))] × 100 (Table 1).

FemaleIBW(Kg):45.5+2.3(height(in)−60)

MaleIBW(Kg):50+2.3(height(in)−60)

**Table 1:** General characteristics.

	Group A (*n* = 90)	Group B (*n* = 106)	*p* value
Gender, *n* (%)	Female: 71 (79%)	Female: 89 (84%)	
	Male: 19 (21%)	Male: 17 (16%)	
Age (years)			
Mean ± SD	43.6 ± 10.1	42.8 ± 7.8	NS
Range	31–58	33–56	
Weight (kg)			
Mean ± SD	86.7 ± 9.8	85.2 ± 8.8	NS
Range	58–106	57–108	
Preoperative BMI (kg/m^2^)			
Mean ± SD	28.6 ± 6.2	27.8 ± 5.8	NS
Range	22.4–32	22.1–35.3	
EWL (%)			
Mean ± SD	35.8 ± 7.2	36.5 ± 7.6	NS
Range	28–103.7	24–98	
Waiting time between BS and PS (years)			
Mean ± SD	3.1 ± 1.7	2.9 ± 1.9	NS
Range	2.1–4.8	2.2–4.6	
Preop-surgery BODY-Q scores			
Mean ± SD	9.77 ± 2.01	9.85 ± 2.06	NS
Range	6.00–15.00	6.00–15.00	

SD, standard deviation; NS, not significant (*p* > 0.05); BMI, body mass index; EWL%, percentage of excess weight loss after the bariatric procedure; BS, bariatric surgery; PS, plastic surgery.

Postoperative follow-up was performed twice a month after discharge for at least 3 months. Postoperative complications were assessed by a senior surgeon and were classified and recorded based on the modified Clavien–Dindo classification (13): postoperative hemorrhage, defined as >200 ml/day in close-suction drainage, transfusion, reoperation for a sharp decrease of hemoglobin; seroma: defined as postoperative fullness and >20 ml of fluid in needle aspiration after the removal of the close-suction drainage; hematoma; fat or dermal necrosis; cellulitis; abscess (positive culture of needle aspiration sample); wound dehiscence; deep vein thrombosis; pulmonary embolism; cardiac or renal complications; readmission to hospital for any reason, except surgical complications. Surgical outcomes, time until removal drain, transfusion rate, wound infection, hospital stay, and complications were compared between the two groups. As a secondary research question, we hypothesized whether Body-Q scores post-surgery in patients who had the new procedure (group B) were increased in comparison with Body-Q scores post-surgery in control subjects (group A). The mean outcome measure was the Body-QTM satisfaction with abdomen score (14), which was administered preoperatively (the day before surgery) and one year postoperatively. Its range is from a minimum of 7 (extreme dissatisfaction in all seven items) to a maximum of 28 (extreme satisfaction in all seven items). The BODY-Q is a condition-specific patient-reported outcome measure that enables a comprehensive assessment of outcomes that are specific to patients undergoing body contouring procedures such as abdominoplasty.

### 2.2 Surgical technique

**Figure 1:**
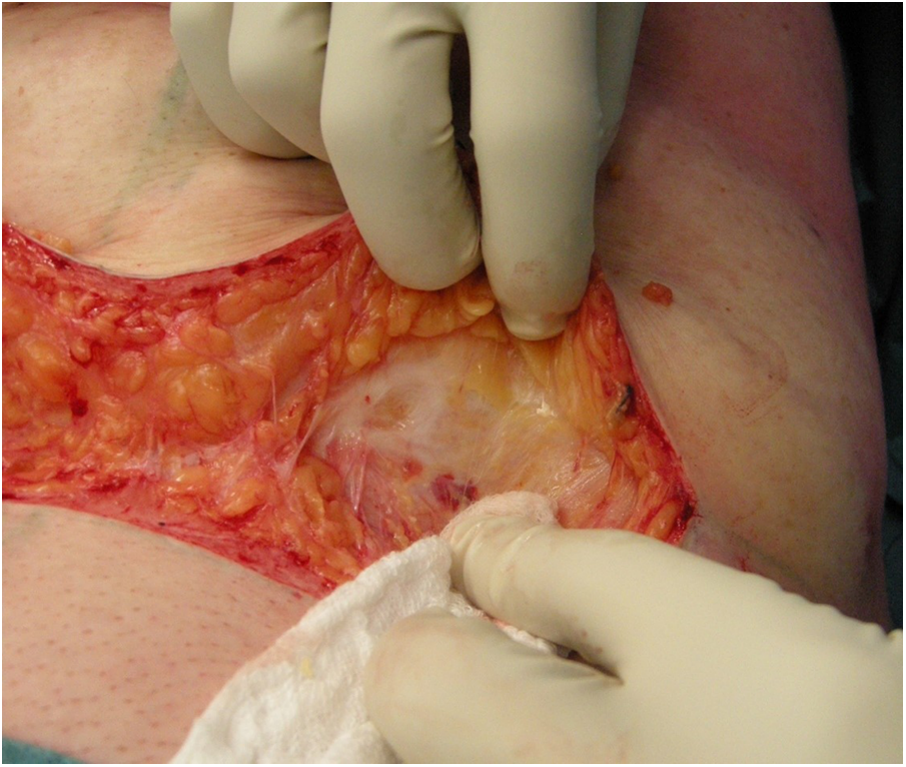
Manual dissection of the skin and exposure of the Scarpa fascia.

**Figure 2:**
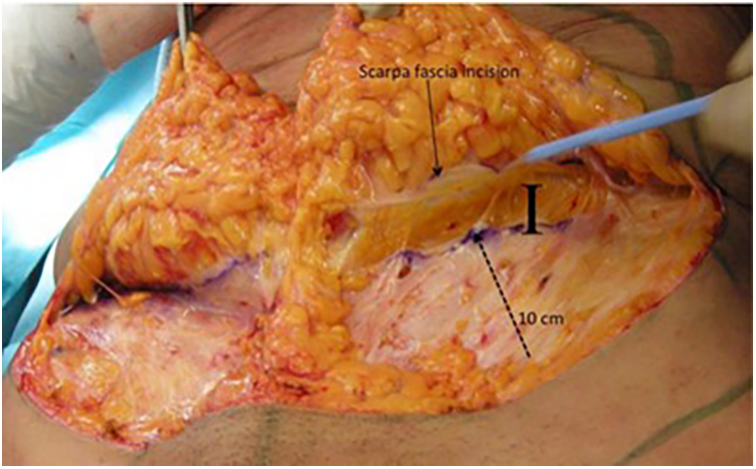
Incision of the Scarpa fascia and the ten cm of the incision skin line (I).

**Figure 3:**
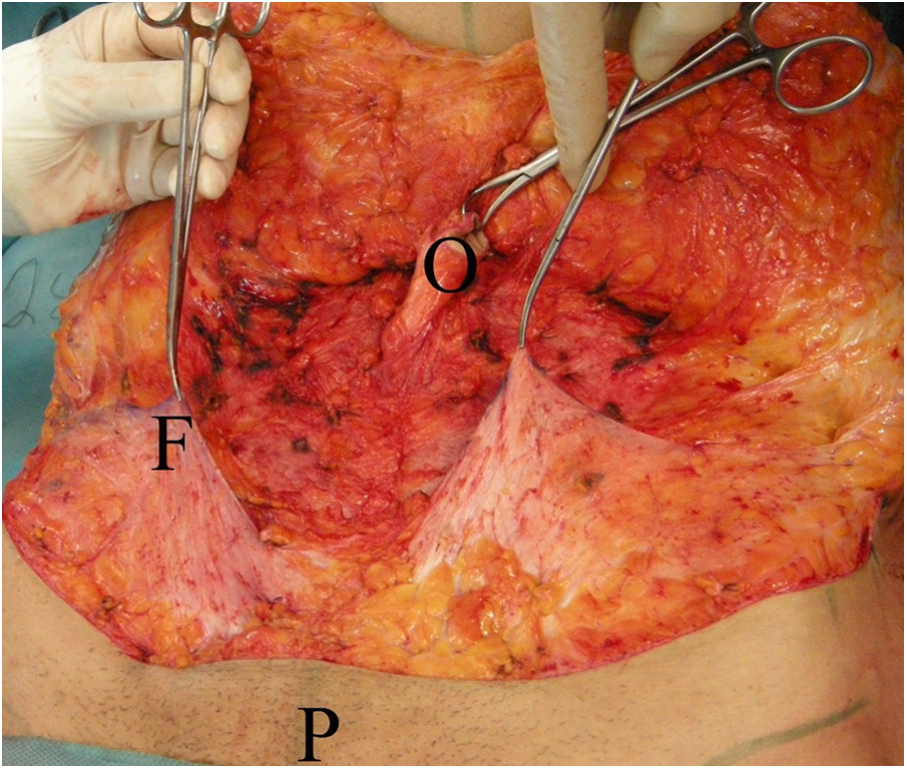
The umbilicus is isolated (O), Scarpa fascia flaps (F) are prepared and pulled up, pubis (P).

Anatomical analysis of the abdomen is performed in the standing position, for identification of the adipocutaneous excess in terms of amount and distribution. In group A, the abdominal flap was dissected in a premuscular plane to the level of the costal margin, as traditionally described. In group B, before surgery, a “W” suprapubic line was drawn on the patient while standing. The line ran between the two anterior superior iliac spines (ASIS), just above the pubic line. A vertical line was drawn from the xiphoid to the pubis. During surgery, the patient was in a supine position with a slight elevation of the legs to avoid excessive tension at the suture line. In group B the first step was infiltration with a solution of 1 mg of adrenaline and 200 mg of lidocaine in 1,000 ml of saline solution, which contributed to reducing arteriolar bleeding and to identifying the Scarpa aponeurosis. The infiltration was performed superficially on the Scarpa fascia as far as the umbilicus and deeper in the supraumbilical region, along the midline. A skin incision was performed reaching the subcutaneous layer. Then, the Scarpa fascia was exposed by manual dissection (Figure 1). The skin was manually separated above the Scarpa fascia to elevate the abdominal flap. If there were fibrotic adherences, we continued the adhesiolysis with a scalpel. Perforating vessels were isolated and ligated with resorbable 2-0 Vicryl stitches. The flaps were undermined medially on both sides as far as the epigastric vessels. Then, the Scarpa fascia was incised above the horizontal incision (Figure 2), and the dissection was performed with a scalpel (Figure 3) in an upward direction, on the muscular fascia up to the umbilicus. The umbilicus was isolated after a periumbilical incision, and the dissection was continued along the middle as far as the xiphoid in an “Eiffel Tower”-shape. The Scarpa's fascia flap was pulled up to a higher position and fixed onto the muscular fascia with absorbable stitches. This traction on the Scarpa fascia flap allowed the elevation of both the inguinal and pubic regions. The umbilicus was reshaped according to the technique described by Cannistrà et al. (15), with one purse string suture positioned along the midline at 12 o'clock at the base of the umbilicus. This maneuver results in downward traction of the supra-umbilical flap, reduces skin tension at the suprapubic incision, and reshapes the natural supraumbilical depression. Two Redon drains (Redon drain CH 10, B. Braun Melsungen AG® Melsungen Germany) were placed and never removed in the first 24 h after surgery. Criteria for removal were drain output per day in each less than or equal to 20 ml over 24 h. The abdominal flap was sutured upon two planes: Monocryl 2-0 (Ethicon®, Johnson & Johnson, NJ, USA) at the level of the fat and subdermal layer while Monocryl 4-0 (Ethicon®, Johnson & Johnson) was used for an intracuticular suture. Our approach allowed for the distribution of skin tension at three distinct levels: on the Scarpa fascia flap is a push-up with an elevation of the inguinal-pubic region, at the level of the umbilicus e with a push-down of the supraumbilical skin, and at the skin edge. Tension was reduced on the abdominal suture, preventing impairment of blood supply, and thus, improving the skin healing process. The mean operative time was about 2 h. The patients were required to wear a moderately compressive abdominal girdle for 3 weeks postoperatively. The patients were usually hospitalized for 3 days and gradually mobilized on the first postoperative day to prevent deep-vein thrombosis.

### 2.3 Statistical analysis

Statistical analyses were performed using the SPSS statistical software (SPSS version 10.0, Inc., and IBM Company, Chicago, Illinois, USA). Continuous data are expressed as means ± standard deviation and range. The chi-square test or Fisher's exact test was used for comparisons between categorical variables and the Student's *t*-test for continuous variables. Patient characteristics are summarized in Table 1. Multivariate analysis was performed using logistic regression analysis (Table 2). All tests were double-sided, and the level of statistical significance was set at *p* < 0.05.

**Table 2:** Outcomes of both groups.

	Group A (90) *n* (%)	Group B (106) *n* (%)	*p* value
Time until removal drain (day)			
Mean ± SD	8.3 **± **2.7	2.4 **± **1.5	<0.05
Range	3.0–12.0	2.0–5.0	
Hospital stay, days			
Mean ± SD	6.3 ±** **3.7	3.6 ± 1.7	<0.05
Range	5.0–12.0	2.0–11.0	
Postoperative complications			
Seroma	16 (17.7%)	5 (4.7%)	NS
Hematoma	8 (8.8%)	2 (1,8%)	NS
Transfusion	6 (6.6%)	1*(0,9%)	NS
Wound infection	7 (7.7%)	3 (2.8%)	NS
Dehiscence/skin necrosis	6 (6.6%)	4 (3.7%)	NS
Abscess formation	5 (5.5%)	2 (1.8%)	NS
Deep venous thrombosis	4 (4.4%)	2* (1.8%)	NS
Pulmonary embolism	1 (1.1%)	0 (0%)	NS

General postoperative data.

* One patient of hematoma group. NS, not significant (*p* > 0.05).

## 3 Results

In this study, 238 abdominoplasties with umbilical transposition were performed at our institute. Forty-two patients were excluded from the statistical analysis because of concomitant correction of ventral hernia, 196 patients were included in the study. The overwhelming majority of patients were women. The 90 patients (45.9%) underwent a laparoscopic Rous-en-Y gastric bypass (LRYGB), 56 patients (28.5%) a laparoscopic sleeve gastrectomy (SG), and the remaining 50 patients (25.5%) a laparoscopic adjustable gastric banding (LAGB). The outcomes in this series are presented in Table 3. There was an average 5-day reduction in drain removal time and a 3-day reduction in length of hospital stay statistically significant difference in group B (*p* < 0.05). Of the 90 patients included in group A, 16 patients (17.7%) presented a postoperative seroma. In group B, which included 108 patients with Scarpa fascia preservation, five patients (4.7%) who developed seroma were aspirated weekly. Three patients required drainage with a needle in two instances, one patient in three instances, and the other in four instances. A trend was found for a lower incidence of seroma in group B with a 73% reduction when compared to group A. Ultrasound screening was performed between the 40–50 post-operative days, however, no late serum was detected in the two groups. Concerning the other complications in group B, four patients (3.7%) presented partial dehiscence/skin necrosis; in one of them revision surgery was needed the others were treated with wet to dry dressing changes until closure. Three patients (2.8%) presented with wound infection treated with antibiotic therapy. Two patients developed hematoma (1.8%); in one of them, a reoperation and transfusion were required. Two patients presented an abscess formation treated with drainage, antibiotic therapy, and wet-to-dry dressing changes until closure. Two patients developed deep vein thrombosis; one of them had undergone reoperation for an important hematoma. One patient in group A developed a pulmonary embolism and was readmitted on the 11th postoperative day. The average postoperative hospital stay was 3.6 days in Group B and 6.3 days in the group A. Additionally, there was no difference in the correlation between the weight of the abdominoplasty removal specimen and the rate of seroma. The rate of seroma was significantly higher in men, those with a BMI > 30 kg/m^2^, and those aged >50 as summarized in the Table 2 (*p* < 0.05). Postoperative Body-Q scores are reported in Table 2. The postoperative score is higher in group B, and the result is statistically significant (*p* > 0.05). The items that improved most in group B were 1 (How your clothes fit your abdomen), 5 (How your abdomen looks in a swimsuit), and 7 (How your abdomen looks when you are naked).

**Table 3:** Multivariate analysis.

Variable	*n*/*N* (%)	*p*	Odds ratio	95% CI for odds ratio	** **
Gender		0.01			
Female	13/160 (8.1%)				
Male	7/36 (19.4%)		2.4	1.9	4.2
Age		0.05			
<40 years	1/45 (0.5%)		0.4	0.2	0.7
40–50 years	12/100 (12%)		1	0.9	1.2
>50 years	7/51 (14.6%)		1.3	1.1	1.9
BMI		0.05			
<25 kg/m^2^	2/38 (5.2%)		0.4	0.2	0.9
25–30 kg/m^2^	8/105 (7.6)		1.2	0.8	1.5
30–32 kg/m^2^	10/53 (18.8%)		2.8	1.9	5.6

One year postoperatively Body-Q scores		
	Group A (*n* = 90)	Group B (*n* = 106)
Mean ± SD	21.50 ± 2.22	23.80 ± 2.32
Range	16.00–25.00	16.00–25.00

Logistic regression predicting likelihood of seroma and/or the need of >3 days closed-sunction drainaige based on gender, age category, and BMI category (n, number of patients with seroma and/or extended drainage; N, number of patients in the group), The postoperative score in group B is significantly higher than in A (*p* < 0.05).

## 4 Discussion

This retrospective study provides evidence that abdominoplasty with Scarpa fascia preservation in post-bariatric patients is clinically important because is associated with a reduction in drain output and consequent early removal of drains, and reduction in hospital stay. Despite the occurrence of complications in both groups, no statistically significant differences were detected. A high rate of complications was reported with the use of the conventional abdominoplasty technique described by Pitanguy in 1964 (16). Savage (17) 1983 noted a 23% incidence of complications in post-bariatric patients. Spieglemann and Levine (18) reported a complication rate of 19%–25%. Seroma is one of the most troublesome complications. Its treatment requires repeated aspirations and the application of elastic compression garments. If the condition becomes chronic and causes a pseudo-bursa, reoperation may be required. As described in various studies, wide undermining is considered one of the main causes of seroma (19, 20). Different approaches have been developed with the intent of decreasing the incidence of seromas. Baroudi and Ferreira (21) and Pollock (22) recommended the placement of numerous quilting sutures, between the undersurface of the adipose tissue and the underlying muscle fascia to eliminate dead space and reduce tension. Despite these recommendations, a high incidence of seroma is still observed after abdominoplasty. Lockwood (23) and Le Louarn and Pascal (24) obtained better results by adding superior and lateral tension. Fibrin glue has also been proposed for collapsing the space below the abdominal flap (25). Khan (26) showed a significant reduction in seroma formation by adding the high-tension procedure to the standard abdominoplasty. In 1999, Vastine et al. (8) reviewed 90 dermolipectomies and noticed that 80% of the population with obesity patients suffered from complications (25% of them being seromas) whereas only 33% of the non-obese people suffered from complications in another study (19). Because of the extent of undermining and the thick abdominal pannus encountered in people with obesity, they are more susceptible to seromas, hematomas, and wound dehiscence. Interestingly, the study by Najera M. et al. (27) showed a higher seroma rate in patients with higher BMI and if liposuction was performed simultaneously (16% vs. 31.2%). We believe that seroma formation is multifactorial and a consequence of lip necrosis secondary to electrosurgery (28) and discontinuation of the Scarpas fascia (29). Blade incisions demonstrated reduced thermal injury depth inflammatory response, and scar width in healing skin compared with electrosurgery. We think that avoiding electrocoagulation prevents lip necrosis, and reduces postoperative local inflammation and consequently, postoperative pain. The acute thermal injury depth is 0 Microm (µm) with scalpel incision and a mean of 763 µm with conventional electrosurgery (30). It is natural that at this depth we have a necrosis of the fat and important liponecrosis with greater inflammation and seroma production. The importance of the Scarpa fascia has been widely described by Lockwood (23) and Saldanha et al. (20) the Scarpa fascia delimits a thin layer of fat, connective tissue, arteries, veins, and lymphatic vessels between the subcutaneous fat and the muscular abdominal plane. Its section at the level of the inguinal ligament, as in other techniques, causes a discontinuation of the lymphatic vessels. The saving of the Scarpa fascia, according to different Authors, would allow the reduction of the volume of drained fluid, the time spent by the drains, and the average hospital stay (31). Many authors recommended the preservation of the Scarpa fascia (22, 23–34) up to the umbilical level; other authors proposed only the preservation of the underlying fascial fat (26, 27). We noticed an anatomical difference between the Scarpa fascia in men and women based on our experience: the fascia in men is thinner and more irregular, so it requires careful handling. Men are also more prone to bleeding. Chong T. et al. (35) also arrived at a similar conclusion whereby after massive weight loss, men are more likely to develop postoperative seroma than women. Using our procedure, the Scarpa fascia flap elevation, in addition to releasing tension on the wound edges, allows an evaluation of the pubis, which is often ptotic in post-bariatric patients. Pubic elevation results in a younger appearance and exposition of the clitoris. Wound closure under skin tension causes great postoperative discomfort, leading to prolonged analgesic administration, longer immobilization, and recovery time. Excessive tension on wound edges can cause tissue hypoxia and is the main reason for the dehiscence or development of hypertrophic scarring. To reduce wound tension during surgery, patients are always positioned with their hips slightly flexed on the surgical table and the sutures are performed upon two different directional tension planes. The importance of preserving lateral perforator vessels was emphasized by Taylor et al. (36) as it avoids healing complications caused by impaired blood supply, which is why we perform an “Eiffel Tower” shaped dissection from the umbilicus to the xiphoid, as described by other authors (20–24). The blood vessels in the abdominal wall in post-bariatric patients are larger than in other patients, this is why we pre-eminently prefer to ligate blood vessels rather than coagulate them. Considering the results of our study, some limitations have to be acknowledged. We conducted a retrospective study, with access only to clinical data that are normally collected within routine clinical practice and with a lack of methodological rigor; in fact, the sample size in the different groups was not homogeneous. Moreover, although the data were prospectively collected, the analysis was retrospective and, therefore, subject to the inherent limitations of retrospective analyses. Nevertheless, our results are consistent with previous studies on this subject and have improved our clinic practice. Applying this technique, it is possible to reduce discomfort for the patient using suction drains and invasive procedures to aspirate seroma. The associated costs of long hospital stays and concomitant medical assistance for complications are also reduced.

## 5 Conclusion

We think that seroma formation can be reduced by safeguarding the fat tissue, avoiding the use of electroctrosection, preserving the anatomy and the continuation of the lymphatic vessels. This procedure could improves the quality of the postoperative course, decreasing surgical complications and achieving better aesthetic results. In our experience we observe that the partial preservation of Scarpa fascia and its push-up fixation during the surgery helps to mold the inguinal-pubic region and reduce skin tension at the level of the suprapubic incision. Such a technique can be easily introduced into a surgeon's practice without interfering with surgical time or general principles of the classic procedure.

## Data Availability

The original contributions presented in the study are included in the article/Supplementary Material, further inquiries can be directed to the corresponding author.
